# Polymorphic mutations in the *polb* gene promoter and their impact on transcriptional activity

**DOI:** 10.1111/1759-7714.14337

**Published:** 2022-02-06

**Authors:** Qingjun Wu, Yuying Qi, Shuanghu Wang, Jian Liu, Peiwu Geng, Quan Zhou, Wenqian Zhang, Jianping Cai, Bin Hu, Dapeng Dai, Hui Li

**Affiliations:** ^1^ Department of Thoracic Surgery Beijing Institute of Respiratory Medicine and Beijing Chao‐Yang Hospital, Capital Medical University Beijing China; ^2^ The Key laboratory of Geriatrics Beijing Institute of Geriatrics, Institute of Geriatric Medicine, Chinese Academy of Medical Sciences, Beijing Hospital/National Center of Gerontology of National Health Commission Beijing China; ^3^ Peking University Fifth School of Clinical Medicine Beijing China; ^4^ Laboratory of Clinical Pharmacy The Sixth Affiliated Hospital of Wenzhou Medical University, The People's Hospital of Lishui Lishui China

**Keywords:** genetic mutation, luciferase reporter system, Polb, promoter, transcription

## Abstract

**Background:**

DNA polymerase β is one of the key enzymes involved in DNA damage repair and its proper expression is strictly controlled within different cells. We previously reported that three genetic mutations in the promoter region of the *polb* gene are prevalent in the Chinese Han population and two types of mutation are associated with thymic hyperplasia. The purpose of this study was to explore whether other mutated sites exist within the promoter region of the *polb* gene.

**Methods:**

Genomic DNAs of 421 healthy Chinese Han individuals were extracted from whole blood samples and used for gene amplification of the promoter region of the *polb* gene. After gel purification, PCR amplicons were sequenced by the Sanger sequencing method and used for sequence alignment with the Lasergene program. PCR products with novel mutations were then subcloned into luciferase reporter plasmid pGL4.10 and transfected into 293T cells for dual‐luciferase activity analysis.

**Results:**

In total, 11 mutated sites were detected in the Chinese Han population and eight of these were reported for the first time. Using a dual luciferase reporter system, it was found that one novel mutation −142 C > G could decrease the transcription activity of the *polb* gene, whereas two novel mutations, −152_−151insC and −218 C > G, could significantly increase the transcription activity of the *polb* gene.

**Conclusions:**

High polymorphic sites could be found in the promoter region of *polb* gene and approximately half of them could influence its transcription activity.

## Graphical abstract

The promoter activity of *polb* is vital for the proper regulation of one important DNA repair enzyme DNA polymerase β. In this study, 11 mutated sites were detected in the *polb* gene promoter in the Chinese Han population and eight were reported for the first time. Further functional analysis revealed that three novel mutations could significantly influence the transcription activity of the *polb* gene. This study expands the overall view of mutations in the *polb* gene promoter and their relationship with the risk of cancer still needs further investigation.

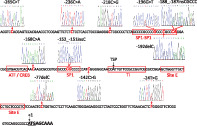

## INTRODUCTION

Base excision repair (BER) is one of the important ways for cells to correct DNA damage and facilitate its repair. It is mainly responsible for repairing damages of DNA based oxidative injury and DNA single‐strand breakage (SSB), caused by reactive oxygen species (ROS) and cytotoxic chemicals (such as alkylation lesions), respectively.^1^ DNA polymerase β *(polb*) is the smallest DNA polymerase in man and is the main DNA polymerase involved in the BER process, with dual‐activities of DNA polymerization and dRP lyase.^2^ During BER, DNA polymerase β can eliminate the dRP group and then fill the gap between the purine or pyrimidine sites that synthesize the injured DNA. In this process, abnormal expression or activity of *polb* will result in the accumulation of DNA lesions.^3^, ^4^ However, because of the relatively lower fidelity of *polb* to other types of DNA polymerases, base mispairing and aneuploidy are likely to be introduced in BER process, especially when the *polb* gene is mutated.^5^, ^6^ Thus, up‐ or downregulated expression of *polb* can cause genetic instability and cellular transformation. Accordingly, it is crucial to maintain the proper regulation and stability of *polb* expression, and both increased or decreased expression will lead to a high risk of cellular mutation.^7^

Previous studies have reported that genetic mutations in *polb* coding gene *polb* have been detected in most common tumors, including colorectal,^8^, ^9^ gastric,^10^ esophageal,^11^ and lung cancers,^12^ etc. In addition, Polb mutants also play a role in promoting cell proliferation, growth, and drug resistance in tumorigenesis. Among the commonly used antitumor drugs, platinum chemotherapies exert antitumor effects by directly damaging DNA, involving monoadducts and DNA cross‐links^13^; Bleomycin (BLM) and doxorubicin (ADM) kill tumor cells by inducing oxidative injury to DNA; other drugs such as temozolomide (TMZ) and melphalan cause genomic DNA alkylation and lead to tumor cell death.^14^ It has been reported that *polb* mutants enhance the repair capacity of tumor cells against these DNA‐damage‐mediated drugs, thus considerably reducing their antitumor effects through the BER pathway.

In addition to mutations in the coding region of *polb* gene, some genetic variations have also been reported in the promoter region of this gene.^15^, ^16^ We recently detected three polymorphic sites in the promoter region of the *polb* gene in the Chinese Han population, two of which were confirmed to be related to thymus hyperplasia. A further functional analysis experiment revealed that −168C > A and −188_−187insCGCCC could significantly impact the transcriptional activity of polb enzymes.^17^, ^18^ However, little is known about whether there are other mutated sites in the *polb* gene promoter region in healthy individuals, or to their impact on the expression of olb protein. In this study, we amplified the fragment of the *polb* gene promoter in 421 healthy Chinese individuals and eight novel mutated sites were discovered after sequence alignment, with nearly half of them having influence on the transcriptional activity of the *polb* gene promoter.

## METHODS

### Subjects

The study subjects were recruited following physical examination of a healthy population in the Beijing Hospital and the People's Hospital of Lishui. The average age of the population was 41.8 years old, of which 209 were male and 212 were female. Informed written consent was obtained from all participants during the blood sample collection process, and this study was approved by the Institutional Ethical Committee of Beijing Hospital.

### DNA extraction and PCR amplification

Genomic DNAs of 421 healthy Chinese Han subjects were extracted from whole blood samples using the magnetic bead method with FineMag Universal Genomic DNA Kit (GENFINE Biotech). Then, 30–50 ng of genomic DNA was used for the preparation of the PCR reaction mixture which included 15 μl 2 × Taq Plus Master Mix II (Vazyme Biotech), 0.2 μmol/l primer pairs (forward primer: 5′‐GGAAACACAATCACCACAACCTT‐3′; reverse primer: 5′‐ACCAGCCTCGATTCTTGCTTT‐3′). PCR amplification reaction was performed according to our previously reported methods^17^, ^18^ and the 1.7 kb amplification product was then separated by 2% agarose gel electrophoresis and purified using a gel recovery kit (Biomed Biotechnology). Purified products were sent to Beijing Tianyi Huiyuan Biotechnology Co., Ltd. for Sanger sequencing on an ABI3730XL DNA Analyzer.

### Sequence alignment

Seqman module of the Lasergene 7.2 program (DNASTAR, Inc.) was used for sequence alignment with the wild‐type human *polb* gene DNA sequence (NC_000008.11) recognized as the template. All chromatogram files were manually inspected and assembled by at least two individuals to ensure their accuracy. Novel mutations were verified with another PCR amplification followed by bidirectional sequencing.

### Dual luciferase plasmid construction

Core promoter region of the *polb* gene (−314 upstream of the start codon with the first base of start codon ATG is defined as +1) was amplified from the PCR products of the wild‐type and mutated *Polb* gene carriers with reported primer pairs (Forward: 5‐AAAA*CTCGAG*CTGGGCTGTCATTCTGAG‐3′, XhoI site was shown as underlined italics; Reverse: 5′‐AAAC*AGATCT*GGCGGCCTGCACCCGAGA‐3′, BglIIsite was shown as underlined italics). Then, the amplicons were gel‐purified and digested with XhoI and BglII enzymes (NEB). After purification, the digested products were subcloned into pGL4.10 vector to yield the recombinant dual‐luciferase plasmid pGL4‐polb.

### Transcription activity determination with luciferase reporter system

We resuspended 293T cells (2 × 10^5^ cells per well on 24‐well plate) which were subsequently transfected with 600 ng pGL4‐polb plasmid and 50 ng pRL‐TK plasmid with liposome 2000 (Invitrogen), following the manufacturer's recommended protocol. Six hours after transfection, cells were cultured with 500 μl fresh DMEM medium containing 10% FBS for additional 48 h. Cell lysates were then prepared with 200 μl passive lysis buffer and 20 μl were used for the Firefly luciferase (Fluc signal) and Renilla luciferase (Rluc signal) activity measurement by dual luciferase reporter assay system on GloMax 20/20 luminometer using the standard detection parameter (Promega). The transcriptional activity of each promoter was calculated with Fluc/Rluc ratio. The ratio of wild‐type *polb* gene promoter was set to 1 and ratios of other mutated *polb* promoters were relative to that of wild‐type and expressed with $\bar{x\;}$ ± SD from three independent experiments. After a student's *t*‐test analysis, *p* < 0.05 was recognized as statistically significant compared with that of wild‐type.

## RESULTS

### Polymorphic mutations in *polb* promoter

**FIGURE 1:**
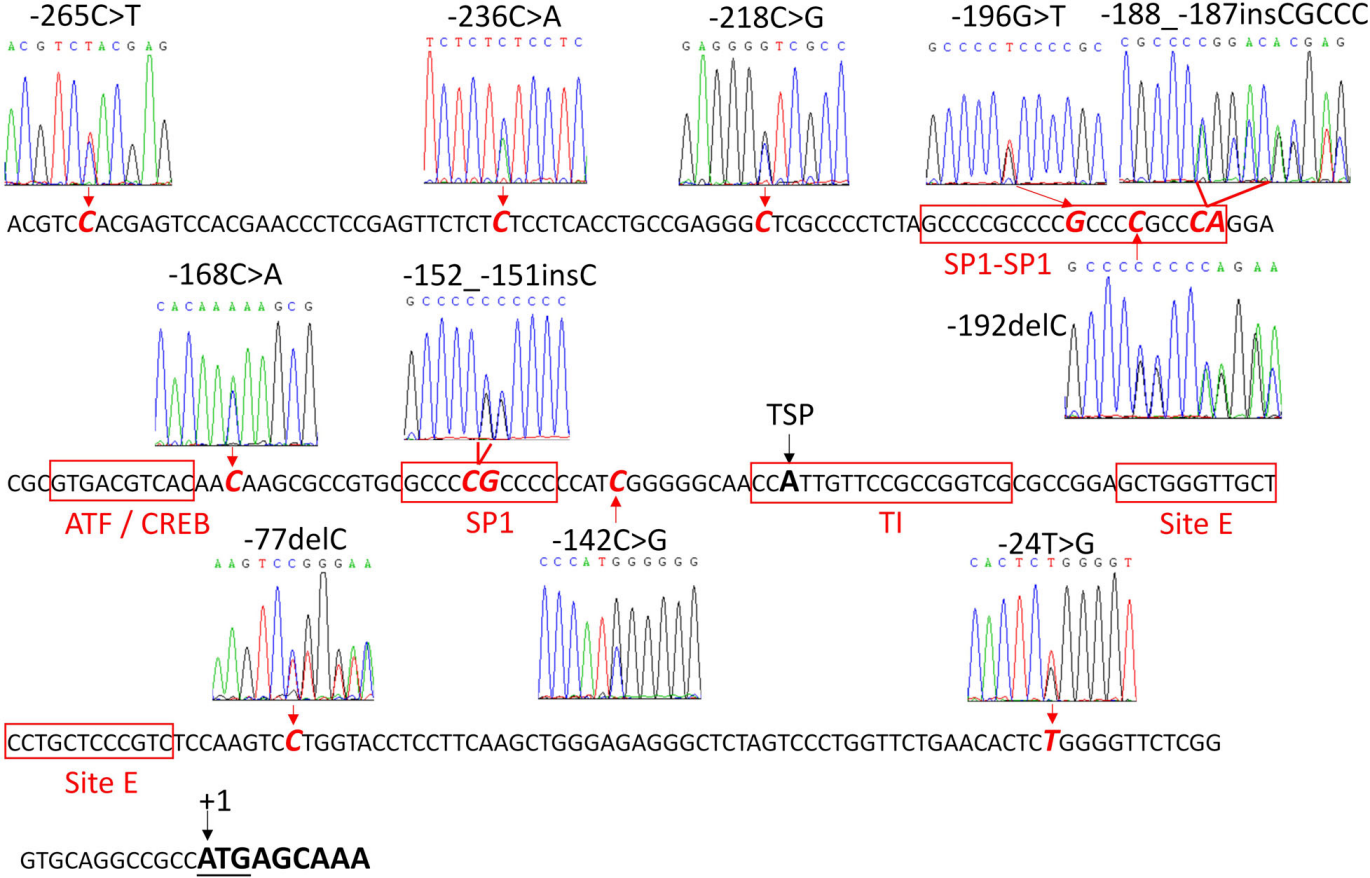
The location of detected mutations in the *polb* gene promoter region. “A” in start codon, bolded and underlined, is designated as the nucleotide +1. Sanger sequencing electropherograms of mutated sites are illustrated above or below the corresponding sites which are emphasized with red bold italic characters. The main elements in the core promoter of the human *polb* gene are boxed and shown in red characters (referring to gene 1995, 164:323–327). TSP: transcription start point. TI: 18‐nt tsp region

To better understand the genetic information about the core promoter region of *polb* gene, 421 healthy Chinese Han individuals were enrolled for DNA sequencing in this study. As a result, 11 different mutated sites in total were detected, eight of which were reported for the first time (Table 1). As illustrated in Figure 1, most of the identified mutations were located within or close to the main elements in the core promoter of the human *polb* gene, indicating they might have some impact on the transcriptional activity of the *polb* gene.

**TABLE 1:** Allele frequencies of the mutations in the *polb* gene promoter of the Chinese Han population

Allele	Number	Frequency (%)
−196G > T	70	8.43
−168C > A	25	2.97
−188_−187insCGCCC	10	1.19
−265C > T	4	0.48
−142C > G	2	0.24
−192delC	2	0.24
−24 T > G	1	0.12
−77delC	1	0.12
−152_−151insC	1	0.12
−218C > G	1	0.12
−236C > A	1	0.12
Wild‐type	724	85.99
Total	842	100.00

### Influence of mutations in the *polb* gene on its transcriptional activity

**FIGURE 2:**
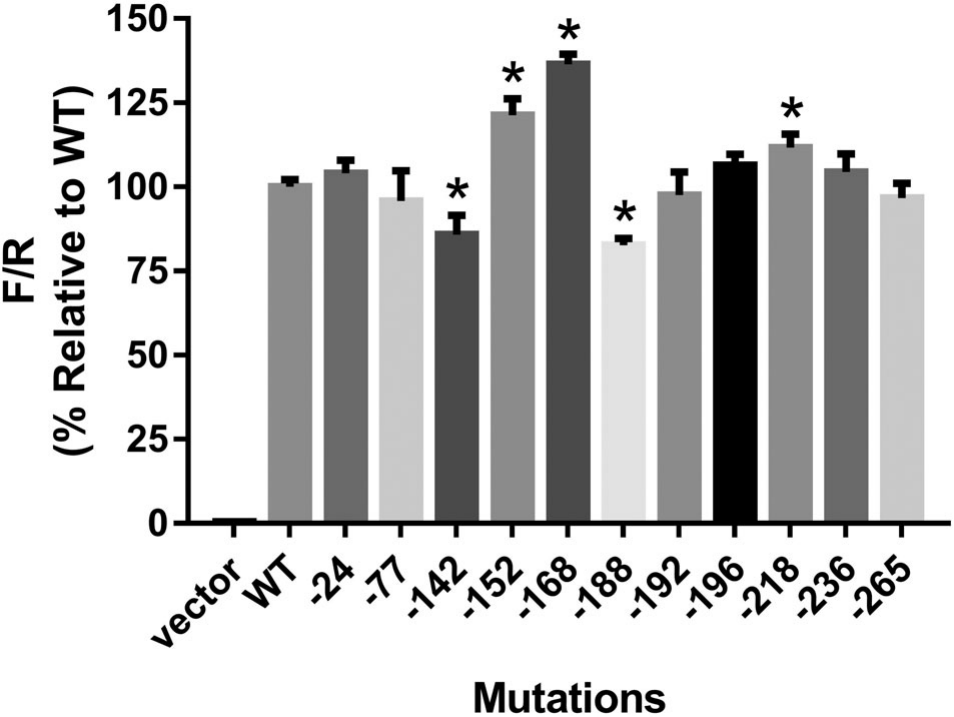
Transcriptional activity analysis of the *polb* gene promoter in 293T cells. *p < 0.05 indicates significant differences from the wild‐type. The ratio of Fluc/Rluc signal of reporter vector containing wild‐type promoter was set to 100% and ratios of signals of vectors containing mutated *polb* promoters were relative to that of wild‐type. Mutated sites are noted in the x‐axis. Each column represented the signals from triple treatments and expressed with $\bar{x}$ ± SD

To explore the biological effects of detected mutations in the *polb* promoter region, dual‐luciferase activity analysis was performed after transfecting the dual‐Glo vectors into 293T cells. As illustrated in Figure 2, three mutations (−152_−151insC, −168C > A and −218C > G) exhibited elevated transcriptional activity and two mutations (−142C > G and −188_−187insCGCCC) showed reduced transcriptional capacity, as compared with that of wild‐type promoter. These data indicate that about half of detected mutations in the human *polb* gene promoter could influence its transcriptional activity in 293 T cells.

## DISCUSSION

DNA structure of the human DNA polymerase β promoter was originally reported by Englander and Wilson in 1990.^19^ In their follow‐up studies, a series of critical regulatory elements were also disclosed in the promoter region of the *polb* gene, which contains an ATF/CRE‐binding site and is short of TATA boxes but rich in GC boxes.^20^, ^21^ Several SP1 binding sites could be found in the GC rich region of the *polb* gene promoter, and these SP1 sites were reported to be vital for the fundamental transcriptional activation.^20^ Similarly, the ATF/CRE‐binding site was likewise proven to play an important role in the activity of the cloned promoter, in which ATF/CREB protein binds to the site and regulates promoter activity via phosphorylation modification (cAMP second messenger pathway).^22^, ^23^

Recently, several studies have reported that mutations in the DNA polymerase β promoter region are abundant in tumors, and these mutations might play a significant role in the regulation of precancerous lesions and tumor progress.^15^, ^16^, ^17^, ^18^ Li et al. reported 11 mutated sites in *polb* gene promoter in human esophageal squamous cell carcinoma tissues with higher occurrence rate than in paracancerous tissues, in which −37C > A (equal to −168C > A in this study) was the hot spot showing elevated transcriptional activity for polb protein.^15^ We previously reported that three polymorphic sites could be detected in both lung cancer tissues and thymic hyperplasia patients, and two mutations had a higher rate in thymic hyperplasia patients than that in normal healthy people.^17^, ^18^ In this study, using 421 healthy Chinese subjects, we also detected 11 different mutated sites in the human *polb* gene promoter. However, only −168C > A (−37C > A in the report by Li et al.) and −196G > T (−65G > T in the report by Li et al.) were also reported. Our functional analysis results indicated that −168C > A exhibited elevated transcription activity compared to that of the wild‐type *polb* promoter, whereas Li et al. reported that both −168C > A and −196G > T could significantly increase the biological activity of the promoter region of the *polb* gene in three different esophageal carcinoma cell lines. We believe that different cell lines might be the main reason for the inconsistency between the study by Li et al. and the present study. Additionally, we reported eight novel mutation sites in the *polb* gene promoter for the first time with three mutations could significantly change the transcriptional activity of promoter, suggesting that polymorphic sites in the polymerase β promoter region were beyond imagination and most of them could impact the expression of Polb protein (Figure 1 Table 1). Specially, most of the mutation sites were located at, or close to, the core element region of the *polb* gene promoter. When expressed in 293 T cells, three mutations (−152_−151insC, −168C > A and −218C > G) exhibited increased transcriptional activity and two mutations (−142C > G and −188_−187insCGCCC) showed reduced transcriptional capacity, as compared with that of wild‐type promoter (Figure 2). These data indicated that sequence changes within or around the core promoter could impact the biological effects of the *polb* gene promoter and further studies are still needed to disclose the molecular mechanisms underlying the regulation of polymerase transcriptional activity by mutation positions, forms, and substitution types.

A number of publications have reported that polymerase β mutants impact on the cell growth, proliferation, and prompt drug resistance of tumor cells, indicating that DNA polymerase β mutants are related to tumor treatment, diagnosis and prognosis.^24^, ^25^, ^26^ In addition, these mutations in the *polb* gene might significantly influence the expression level of Polb protein in cancer cell lines and cancer tissues, and up‐ or downregulated expression of Polb could cause genetic instability and cellular transformation.^12^, ^27^, ^28^ In this study, we found that five mutations in the *polb* gene promoter could induce or reduce its transcriptional activity, indicating that attention should be drawn to the correlation between DNA polymerase β promoter mutations and the risk of cancer in healthy people, as well as the interaction between the polymerase β promoter region mutations and coding region mutations and their synergistic effect on tumorigenesis in the future. Long‐term follow‐up investigation and large‐scale genetic screening in cancer tissues are also needed to elucidate the influence of detected mutations on the incidence of cancer.

In conclusion, a total of 11 mutated sites were detected in a healthy Han Chinese population, among which eight were newly discovered for the first time. The transcriptional activity of the *polb* gene could be reduced by −142C > G, whereas the transcriptional activity was improved by −152_−151insC and −218C > G. Our study significantly expands the overall view of mutations in the *polb* gene promoter and their relationship with the risk of cancer in healthy individuals still needs further investigation.

## CONFLICT OF INTEREST

The authors have no conflicts of interest and no disclosures to declare.
